# Inulin-lipid hybrid (ILH) microparticles promote pH-triggered release of rifampicin within infected macrophages

**DOI:** 10.1007/s13346-022-01287-3

**Published:** 2023-01-11

**Authors:** Sajedeh Maghrebi, Nicky Thomas, Clive A. Prestidge, Paul Joyce

**Affiliations:** UniSA Clinical and Health Sciences, University of South Australia, Adelaide, South Australia 5000 Australia

**Keywords:** Inulin, Lipid-based delivery, pH-responsive, Macrophage, Rifampicin, Intracellular bacteria, *Staphylococcus aureus*

## Abstract

Intracellular bacteria serve as a problematic source of infection due to their ability to evade biological immune responses and the inability for conventional antibiotics to efficiently penetrate cellular membranes. Subsequently, new treatment approaches are urgently required to effectively eradicate intracellular pathogens residing within immune cells (e.g. macrophages). In this study, the poorly soluble and poorly permeable antibiotic, rifampicin, was re-purposed via micro-encapsulation within inulin-lipid hybrid (ILH) particles for the treatment of macrophages infected with small colony variants of *Staphylococcus aureus* (SCV *S. aureus*). Rifampicin-encapsulated ILH (Rif-ILH) microparticles were synthesized by spray drying a lipid nano-emulsion, with inulin dissolved throughout the aqueous phase and rifampicin pre-loaded within the lipid phase. Rif-ILH were strategically designed and engineered with pH-responsive properties to promote lysosomal drug release upon cellular internalization, while preventing premature rifampicin release in plasma-simulating media. The pH-responsiveness of Rif-ILH was controlled by the acid-mediated hydrolysis of the inulin coating, where exposure to acidic media simulating the lysosomal environment of macrophages triggered hydrolysis of the oligofructose chain and the subsequent diffusion of rifampicin from Rif-ILH. This pH-provoked release mechanism, as well as the ability for ILH microparticles to be more readily internalized by macrophages, was found to be influential in triggering a 2.9-fold increase in intracellular rifampicin concentration within infected macrophages, compared to the pure drug. The subsequent increase in exposure of intracellular pathogens to rifampicin leads to a ~ 2-log improvement in antibacterial activity for Rif-ILH, at a rifampicin dose of 2.5 µg/mL. Thus, the reduction in viability of intracellular SCV *S. aureus*, in the absence of cellular toxicity, is indicative of ILH microparticles serving as a unique approach for the safe and efficacious delivery of antibiotics to phagocytic cells for the treatment of intracellular infections.

**Graphical Abstract:**

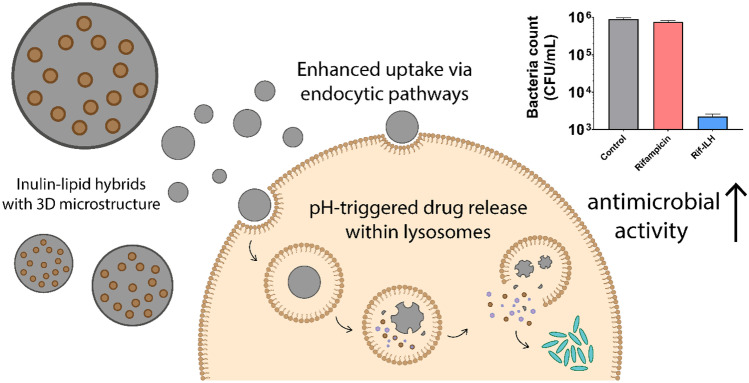

## Introduction

The over-consumption and over-prescribed application of antibiotics has triggered the emergence of drug-resistant bacteria that have evolved an array of evasive mechanisms. For example, in 2011, the World Health Organization (WHO) reported that among the 12 million worldwide cases of tuberculosis, 630,000 of these patients have developed a multi-drug resistance strain [1]. Among the various evasive mechanisms, many bacterial pathogens, including *Mycobacterium tuberculosis*, *Staphylococcus aureus* and *Salmonella enterica*, are now capable of being shielded within host cells, which leads to latent or recurrent infection [2, 3]. Intracellular pathogens shelter and live mostly within the mononuclear phagocyte system (MPS), comprised of phagocytic cells, such as blood monocytes and tissue macrophages. The main role of the MPS is to clean the blood stream by engulfing and killing foreign particles/micro-organisms by forming phagolysosomes that digest foreign particles/micro-organisms [4]. However, intracellular bacteria are capable of ‘hijacking’ the signalling pathway in order to live in a host cell’s environment and hence use macrophages as a sanctuary [5, 6]. This reservoir allows pathogenic agents to establish secondary infectious foci and leads to the recurrence of systemic infections [7, 8].

The intracellular localization of pathogens provides a situation where bacteria are protected from the action of antibiotics [9]. Antibacterial agents belonging to the β-lactam and aminoglycoside families have limited penetration to the host cells due to their high hydrophilicity [10]. Furthermore, fluoroquinolones and macrolides exhibit restricted and relatively low intracellular retention, despite their ability to penetrate rapidly across cellular membranes [11]. To compensate for the low drug concentration at target sites, high doses of antibiotics are frequently prescribed, which further contributes to antibiotic resistance. Despite scientific advancements, less than one-third of prescribed antibiotics exert any activity against intracellular pathogens [1]. In the past 5 years, less than 50 new antimicrobial compounds have progressed to the final stages of clinical trials (i.e. phase II/III human trials), with only 15 new therapeutics receiving regulatory approval [12]. Of the approved antibiotics, none has been specifically designed for treating intracellular bacteria and as such, there is no guarantee that these conventional treatments will lead to improved bacterial eradication. Thus, it is expected that bacteria will continue acquiring resistance unless (1) new therapeutics that are capable of efficient cellular internalization are designed [13] and/or (2) formulation and delivery strategies for antimicrobial molecules are improved that promote their cellular uptake and co-localization with the infection site [14].

A promising approach for the treatment of intracellular infections is to repurpose existing antibiotics via their encapsulation within micro/nano-carriers designed for increasing cellular membrane penetration and uptake [15–17]. This particle drug delivery approach mimics the endocytic or phagocytic pathway of pathogens to localize within the MPS [18, 19]. By using micro/nano-carriers to efficiently deliver the drug to the infection site, the amount and frequency of dosage can be reduced, thus reducing systemic exposure to the antibiotic. In doing so, the risk of triggering antibiotic resistance is lowered and toxicities relating to therapy are prevented [20, 21].

One such potential drug delivery vehicle that can be used to transport antibacterial drugs to intracellular site of infections is inulin, a biocompatible and biodegradable polysaccharide [22, 23]. Owing to its unique immunomodulatory properties through indirect and direct mechanisms (e.g. inducing pro- and anti-inflammatory cytokines, detection by dendritic cells through receptor ligation of pathogen recognition receptors) [24, 25], inulin has been successfully used as a vaccine adjuvant for bacterial, viral, protozoan antigens, as well as in anticancer treatment [26–28]. Recent studies have highlighted the ability to covalently attach drug molecules to the oligofructose chain of inulin in order to transport antibacterial agents directly to phagocytes infected with intracellular pathogens, whereby the use of pH-dependent drug conjugates introduces the ability for pH-mediated drug release once internalized through phagocytic pathways [29–31]. Furthermore, Afinjuomo et al. showed that freeze-dried inulin microparticles have excellent tropism towards phagocytic cells, and an ability to bind and be internalized by monocytes with high efficiency [29, 30]. However, the reliance of biolabile conjugates between the inulin chain and drug molecules introduces a number of fundamental challenges that restricts the clinical and commercial translatability of inulin-based drug delivery vehicles. Limitations associated with this approach include (i) alterations to the uptake behaviour of inulin due to changes in the size, charge and morphology of the polysaccharide chains, (ii) resource- and labor-intensive synthesis approaches and (iii) the potential formation of alternate metabolites in vivo, due to limited control over the drug-conjugate cleaving mechanisms, which requires additional approval from regulatory bodies.

Subsequently, an alternate approach is required to harness the potential of inulin for the delivery of antibiotics to intracellular infections. We recently developed polymer-lipid hybrid (PLH) microparticles for the delivery of rifampicin to macrophages infected with intracellular small colony variants of *Staphylococcus aureus* (SCV *S. aureus*) [16]. To synthesize PLH microparticles, a PLGA-nanoparticle stabilized lipid emulsion was spray dried to create hybrid microparticles where lipid was encapsulated within a three-dimensional polymeric matrix. It is suggested that a parallel approach can be applied to create an inulin-lipid hybrid (ILH) drug delivery vehicle with the ability to (i) take the advantage of the pH-responsive characteristics of inulin to provide pH-mediated drug release within the intracellular environment of macrophages and (ii) to eliminate the need for biolabile conjugates between the inulin chain and drug molecules by encapsulating the drug within the lipid nano-droplet phase of ILH (particularly relevant for poorly soluble/lipophilic antimicrobials).

Thus, the present study aimed to develop a pH-sensitive drug delivery system composed of lipid nano-droplets coated with inulin in a dry powder form where rifampicin (a model poorly soluble antimicrobial) was encapsulated as an antimicrobial agent. The ability of ILH microparticles to effectively transport rifampicin to the site of infection was systemically investigated by monitoring drug release and uptake within the intracellular environment of macrophages and the subsequent reduction of SCV *S. aureus* colony forming growth.

## Materials and methods

### Materials

Capmul MCM was obtained from Abitec Pty Ltd (NSW, Australia). Inulin from dahlia tubers (with a degree of polymerization of 35), rifampicin, Nile red, phosphate-buffered saline (PBS), deuterium oxide (D_2_O) Dulbecco’s Modified Eagle’s Medium (DMEM), 4’,6-diamidino-2-phenylindole (DAPI), penicillin–streptomycin antibiotic mixture and Fetal Bovine Serum (FBS) were purchased from Sigma-Aldrich (Castle Hill, Australia). The following materials were of analytical grade and obtained from Ajax Finechem Pty Ltd., Australia: acetonitrile, acetone, dichloromethane and methanol. Murine macrophages RAW 264.7 were obtained from American Type Culture Collection (ATCC, Manassas, VA, USA). Small colony variants (SCV) of *S. aureus* were provided by Dr. Stephen Kidd (University of Adelaide, Australia).

### Synthesis of rifampicin-loaded inulin-lipid hybrid microparticles

Rifampicin-loaded ILH (Rif-ILH) microparticles were synthesized using a two-step homogenization and spray-drying process. Firstly, a 2% w/v lipid-in-water emulsion was prepared by dispersing Capmul MCM (1 g) and rifampicin (100 mg) in Milli-Q water (50 mL), with stirring at 1000 rpm for 30 min at room temperature. The emulsion was ultrasonicated for a further 30 min to create a nano-emulsion. An inulin solution (2% w/v), prepared by dissolving inulin (1 g) in Milli-Q water (50 mL) with stirring and heating at 60 °C for 15 min, was added dropwise to the homogenized emulsion and stirred for another 1 h. The nano-emulsion, with inulin dissolved in the aqueous phase, was spray dried (Mini Spray Dryer B-290, Büchi Labortechnik AG) to form ILH microparticles under the following conditions: emulsion flow rate: 0.5 mL/min, air flow rate: 0.6 m^3^/min, inlet temperature: 160 °C, outlet temperature: 90 °C and an aspirator setting of 100%.

Rifampicin-loaded lipid micro-droplets (Rif-lipid) were also prepared to serve as a control system to Rif-ILH. To achieve this, rifampicin (100 mg) was dissolved in Capmul MCM (1 g), followed by addition of 50 mL Milli-Q water. The emulsion was then stirred at 1000 rpm for 30 min to create lipid micro-droplets. Note: No homogenization process was applied in an effort to form lipid micro-droplets with a comparable particle size to ILH microparticles.

### Characterization of ILH microparticles

#### Size and zeta potential

Particle size was evaluated by using laser diffraction (Malvern Mastersizer, Malvern Instruments, Worcestershire, UK), where samples were dispersed in PBS (pH 7.4) with stirring at 300 rpm for 30 min at room temperature prior to the measurement. Zeta potential was measured using phase analysis light scattering instrument (Zetasizer Nano ZS, Malvern Instruments, Worcestershire, UK) with the laser set at a 173° fixing scattering angle. Spray-dried ILH microparticles were dispersed in PBS (pH 7.4) at a concentration of 0.1 mg/mL w/v and subsequently the average particle size was measured (refractive index = 0.145). All measurements were performed in triplicate.

#### Scanning electron microscopy (SEM)

SEM (Carl Zeiss Microscopy, Oberkochen, Germany) was used to observe the morphology of the prepared ILH particles. A sample of ILH was placed on carbon tape and sputter-coated using a thin layer of platinum (~ 10–20 nm) prior to imaging. The SEM imaging was performed using accelerating voltages of 1–2 kV.

#### Quantification of drug loading

Rifampicin loading within ILH microparticles was determined using extraction and analysis by high-performance liquid chromatography (HPLC) (UFLC XR, Shimadzu, Japan). Approximately 10 mg of powder was weighed and dissolved in 10 mL methanol:water (50:50) mixture followed by sonication for 45 min. Samples (1 mL) were then centrifuged for 15 min at 8944 g and the supernatants were collected and properly diluted prior to be analyzed using HPLC, under the following conditions: mobile phase: mixture of methanol, phosphate buffer (pH 7.5) and acetonitrile (50:33:17%, v/v); stationary phase: C18 column (25 × 0.46 cm internal diameter, 5 μm pore size, Altech); flow rate: 1 mL/min; detection wavelength: 230 nm.

### Assessing pH-responsiveness from ILH microparticles

#### In vitro drug release

The pH-responsive nature of ILH microparticles was evaluated in vitro within neutral media (PBS; pH 7.4), simulating the pH environment of plasma, and artificial lysosomal fluid (ALF; pH 4.5), simulating the acidic environment inside macrophages. ALF was prepared according to the composition previously described in the literature [32]. ILH was dispersed in PBS or ALF (10 mL, 37 °C) at a concentration equivalent to 80 µg/mL rifampicin with constant stirring at 200 rpm for a 4-h period. Aliquots (500 μL) were taken periodically and were immediately centrifuged (10 min, 8944 g). The supernatant was collected and diluted with mobile phase prior to HPLC analysis for quantification of drug release, while an equal volume of fresh media was used to re-disperse the pellet before being transferred back into vials. All samples were evaluated in triplicate.

#### In vitro cleavage and breakdown of inulin

Proton nuclear magnetic resonance (^1^H NMR) spectroscopy was employed to determine changes in the degree of polymerization (DPn) of the inulin chain throughout in vitro release studies by modifying the protocol developed by Barclay et al. [33]. Briefly, ILH microparticles were dispersed in PBS (pH 7.4) or ALF (pH 4.5) as above. Aliquots (500 μL) were taken periodically and immediately neutralized by addition of 30 μL saturated sodium bicarbonate solution (in the case of ALF), to inhibit further acid-mediated inulin hydrolysis, prior to centrifugation (10 min, 8944 g). The pellet was redispersed in D_2_O (1 mL) for ^1^H NMR studies, where the spectra were analyzed by setting the integral for the anomeric glucose proton at 5.44 ppm to 1 and integrating this peak against the representative peaks for glucose and fructose protons between 3.30 and 4.40 ppm (Integral X). Inulin DPn was calculated using Eq. (1) below:

1 $$\mathrm{DPn}= \frac{\mathrm{Integral }\;X-6}{7}+1$$

Two ^1^H NMR spectra were obtained for each sample, with analysis on each spectrum performed in triplicate to ensure consistent spectral integration. DPn values are reported as mean ± S.D. based on this analytical approach [33].

Time-dependent changes in ILH particle size were quantified in PBS (pH 7.4) and ALF (pH 4.5) using laser diffraction (Malvern Mastersizer, Malvern Instruments, Worcestershire, UK) with a refractive index of 0.145.

### Cellular cytotoxicity of ILH microparticles

A cytotoxicity study was conducted in RAW 264.7 macrophages using the previously reported method [34]. One hundred microliters of cell suspension at a density of 7000 cells per well was seeded in 96-well plate and incubated (37 °C, 5% CO_2_) for 12 h to allow the attachment of cells. After incubation, the supernatant was discarded and replaced with 200 µL of particle solution (in DMEM) at various concentrations (ranging from 10 to 100 µg/mL) and incubated for further 24 h. After incubation, the cells were washed with sterile PBS and 10 µL of MTT (3-(4,5-dimethylthiazol-2-yl)-2,5-diphenyltetrazolium bromide) solvent was added to each well prior to incubation for a further 4 h. Afterward, the supernatant was discarded, and the absorbance was read at OD = 540 nm using a microplate reader.

### Particle uptake studies in macrophages

#### Uptake studies using flow cytometry

The cellular uptake of ILH particles and lipid micro-droplets was investigated using fluorescence-activated cell sorting (FACS) [34]. Freshly grown RAW 264.7 (~ 7 × 10^4^ cells per well) were plated in a 24-well plate and incubated for 1 day to promote cell adherence. After supernatant removal, the cells were exposed to either Nile red (50 µg/mL in DMEM) or Nile red–loaded formulations at a final concentration equivalent to 50 µg/mL. Following 1-h and 4-h incubation, the cells were thoroughly rinsed (three times using sterile PBS) and collected from each well of plate. The cells were centrifuged at 600 g for 5 min and the subsequent cell pellets were resuspended in FACS buffer. The Nile red intensity inside cells was measured using Accuri C6 Plus flow-cytometer (BD Biosciences, Franklin Lakes, New Jersey, USA).

#### Uptake studies using confocal microscopy

The uptake of particles by RAW 264.7 cells was visualized using confocal laser scanning microscopy (CLSM; Zeiss Elyra PS.1 laser scanning confocal microscope, Germany). Briefly, cells were incubated overnight prior to being exposed to free Nile red, Nile red–loaded lipid micro-droplets and ILH at a Nile red concentration of 50 µg/mL for 1 h. After incubation and prior to staining, the nuclei and cytoskeleton with 4′,6-Diamidino-2-Phenylindole (DAPI) and Alexa-488, respectively, the cells were rinsed with PBS three times and fixed with paraformaldehyde (PFA) (4%) for 20 min. DAPI with the emission wavelength of 461 nm (excitation wavelength 358 nm) and Alexa- 488 with 525 nm emission wavelength (excitation wavelength 490 nm) appeared as blue and green, respectively.

### Rifampicin internalization within macrophages

To study the intracellular rifampicin uptake, RAW 264.7 cells at density of 5 × 10^4^ cells were plated in each well of a 96-well plate using fresh DMEM and incubated overnight. After incubation, the supernatant was discarded and the attached cells were washed three times with PBS, prior to treating the cells with an 80 µg/mL rifampicin dose (samples were suspended in DMEM) for 1-h and 4-h incubation periods. Following this, the cells were washed 4 times to ensure the removal of extracellular particles and lysed for 15 min with DMSO at 37 °C, 5% CO_2_. The lysed cells were then collected in an Eppendorf tube and centrifuged for 5 min at 600 g. The intracellular rifampicin uptake was evaluated by analyzing the supernatant using described HPLC method.

### In vitro antibacterial studies

#### Intracellular bacterial staining

To confirm and visualize the existence of SCV *S. aureus* inside macrophages, RAW 264.7 cells were seeded in a 6-well culture slide at a density of 2.5 × 10^5^ cells per well and cultured overnight. RAW 264.7 cells were then infected with bacteria at a density of 2.5 × 10^7^ cells/well followed by 1-h incubation. Following incubation, macrophages were washed 4 times with PBS to remove the extracellular bacteria and then fixed with 4% PFA. Prior to imaging by confocal microscopy, the nuclei were stained with DAPI and the cell/bacteria membrane was stained with Alexa-488.

#### In vitro efficacy studies against intracellular SCV *S. aureus*

The intracellular infection assay was performed using the method previously reported [34]. Briefly, RAW264.7 cells were grown in a 24-well plate at a seeding density of 1 × 10^5^ cells/mL for 24 h at 37 °C, 5% CO_2_. An overnight culture of *S. aureus* SCV WCH-SK2, a stable clinical isolate that does not revert to the parent strain upon subculturing, was prepared in tryptic-soy broth (TSB) and diluted to a multiplicity of infection (MOI) of 10:1 in DMEM media (without FBS) [35]. The bacterial suspension was added to the cells and incubated for 1 h. Following incubation, extracellular bacteria were removed and washed thrice and the infected cells were treated with rifampicin solution (in DMEM media), rifampicin-loaded ILH (Rif-ILH) and lipid micro-droplets (Rif-Lipid) at rifampicin concentration of 0.5 and 2.5 μg/mL for 4 h. Following this, cells were washed extensively and lysed with 0.1% Triton-X to harvest intracellular bacteria. Bacterial suspension was serially diluted and plates on TSA for incubation at 37 °C for 24 h. The Colony Forming Unit (CFU) was then calculated based on the number of grown bacteria on tryptone soy agar (TSA) plate.

## Results and discussion

### Fabrication and characterization of rifampicin-loaded inulin-lipid hybrid microparticles

**Fig. 1:**
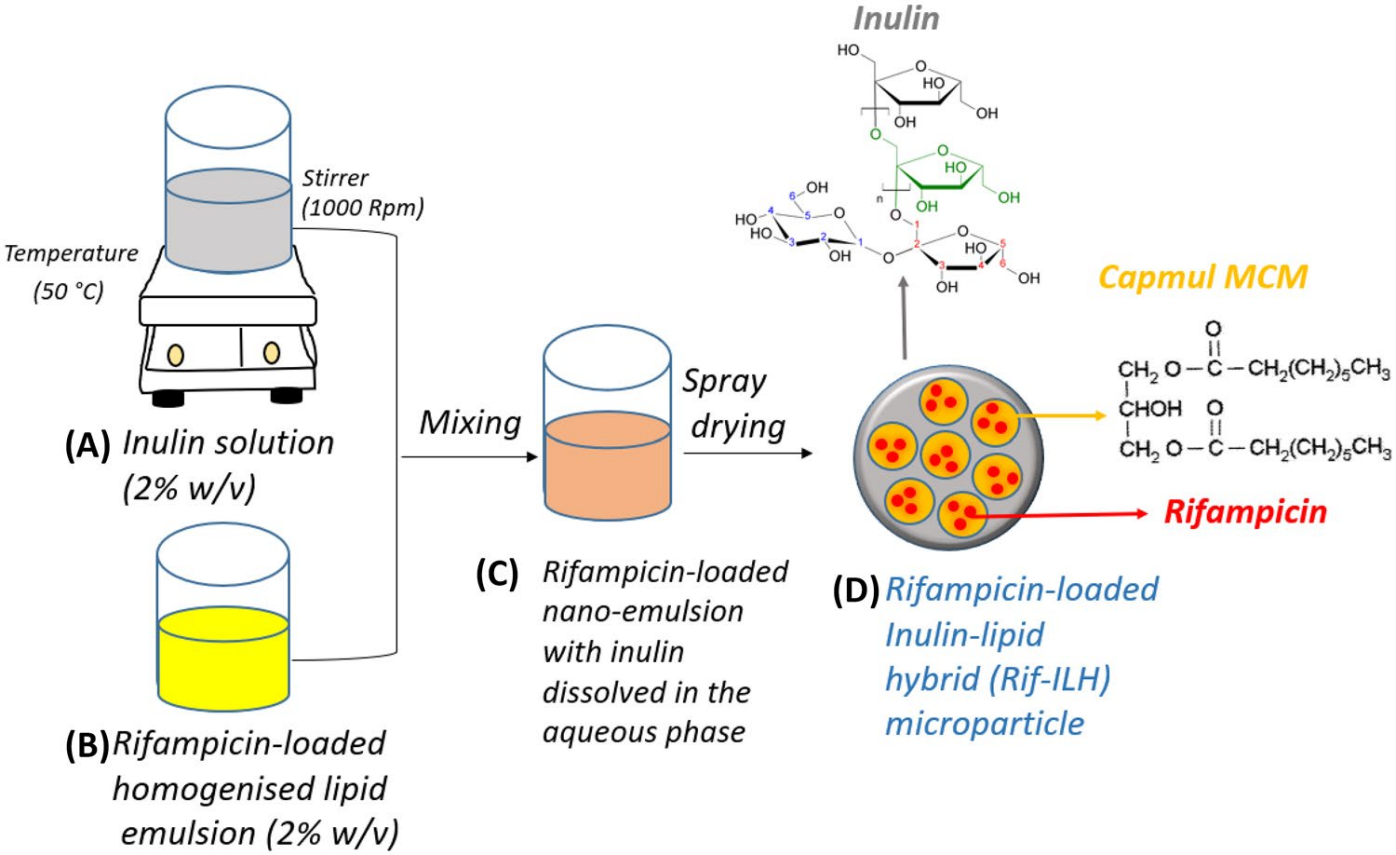
Schematic representation of the two-step fabrication process for Rif-ILH microparticles: **A** an inulin solution and **B** a rifampicin-loaded homogenized lipid emulsion are **C** combined with mixing and **D** spray dried to form Rif-ILH microparticles

Rif-ILH microparticles of 3.11 ± 1.1 µm mean particle size were synthesized by spray drying rifampicin-encapsulated lipid nano-droplets (diameter = 160 ± 27 nm), comprised of medium chain length lipids dispersed within an inulin aqueous solution at a 1:1 lipid:inulin ratio (Fig. 1). Rif-ILH microparticles revealed a negative zeta potential of − 11.5 ± 0.53 mV, which was attributed to deprotonation of inulin hydroxyl groups when exposed to aqueous media [36]. Rifampicin-loaded lipid micro-droplets (Rif-lipid), 2.46 ± 0.8 µm in diameter with a zeta potential of − 4.18 ± 1.2 mV, were also synthesized to serve as a control system to Rif-ILH. Rifampicin loading within Rif-ILH and Rif-lipid was 5.32 ± 0.84% (w/w) and 9.12 ± 1.10% (w/w), respectively.

**Fig. 2:**
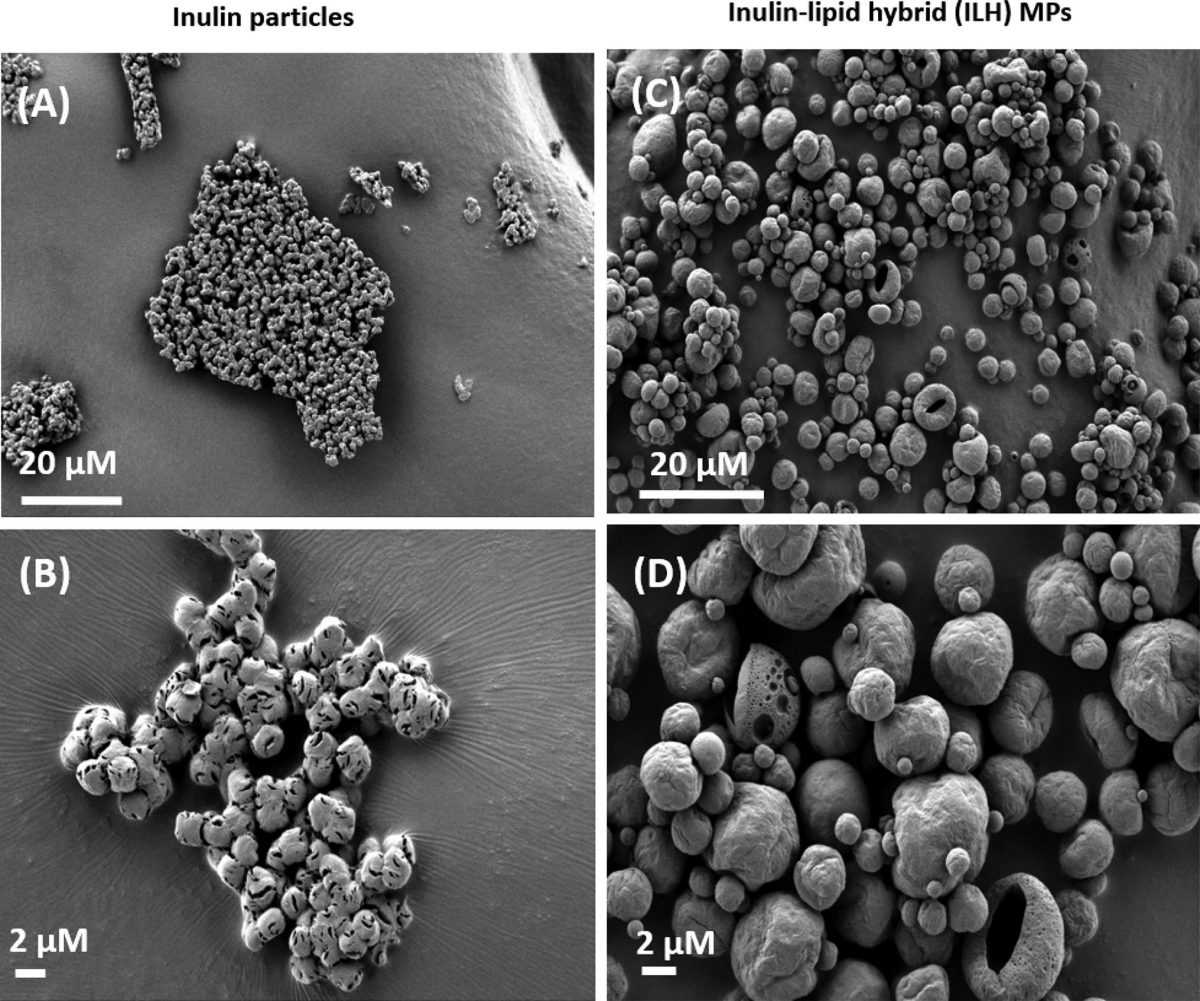
SEM images of dry powder aggregates of raw inulin (**A** and **B**) and rifampicin-loaded inulin-lipid hybrid (Rif-ILH) microparticles (1:1 lipid:inulin ratio) (**C** and **D**)

The morphology of powdered inulin particles and spray-dried Rif-ILH microparticles was investigated using SEM. As shown in Fig. 2, raw inulin powder comprised 1–2 µm particles with a spherulite-like discoid shape, which consists of stacks of lamellar sheets, according to previous investigations [6]. In contrast, Rif-ILH microparticles were mainly between 2 and 5 µm in diameter, of spherical shape and a smooth surface morphology. It is anticipated that inulin accumulated at the surface of Rif-ILH particles, with lipid nano-droplets being encapsulated within a three-dimensional inulin matrix, comparable to alternate polymer-lipid hybrid microparticles prepared through an equivalent fabrication approach [37].

### In vitro rifampicin release studies

**Fig. 3:**
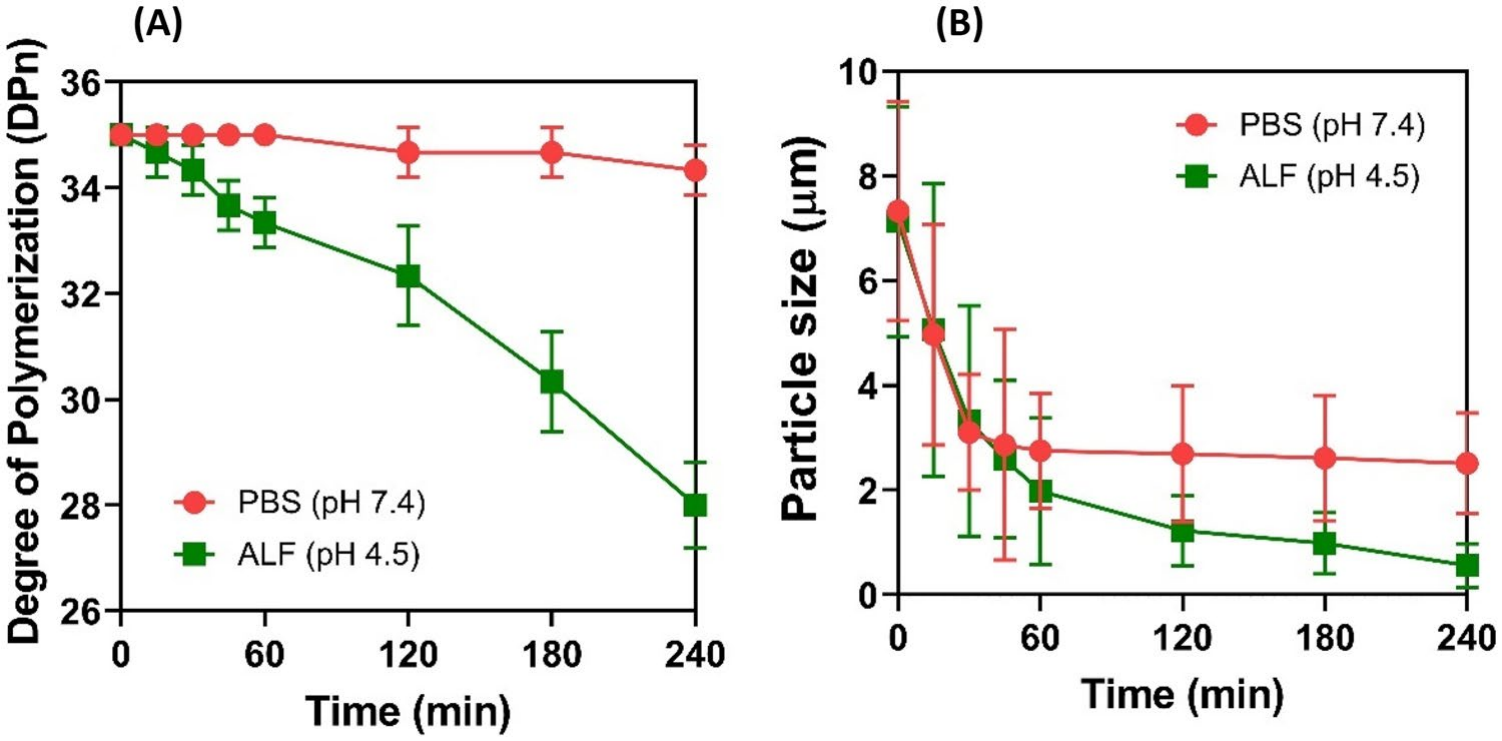
The impact of media composition on inulin degradation within ILH microparticles, evidenced through time-dependent changes in **A** average degree of polymerization (DPn) of the inulin chain, and **B** particle size of ILH microparticles. Data is represented as mean ± SD (*n* = 3)

ILH microparticles were strategically designed with pH-responsive properties, through the inclusion of acid-labile inulin within the shell of the hybrid particles, so that triggered release of the encapsulated cargo is prompted through particle internalization within the acidic environment of macrophage lysosomes. To assess the degradation behaviour of ILH microparticles in biologically relevant media simulating the neutral extracellular environment and acidic lysosomal environment, ILH particles were dispersed in PBS (pH 7.4) and artificial lysosomal fluid (ALF, pH 4.5) [10], respectively. The degree of polymerization (DPn) of the inulin chain and particle size was monitored in a time-dependent manner, which highlighted key differences in particle behaviour between the two media environments (Fig. 3). That is, the inulin fructooligosaccharide chain was stable under neutral conditions, with only a minor reduction in chain length from DPn = 35.0 to DPn = 34.3 ± 0.5 being observed over the 4-h dispersion period. In contrast, the inulin chain was hydrolyzed in acidic ALF media, as evidenced by a reduction in DPn from 35.0 to 28.0 ± 0.8 after 4 h. pH-mediated hydrolysis corresponded with a greater reduction in particle size for ILH when dispersed in ALF, compared to PBS, where the mean particle size decreased to 0.56 ± 0.2 µm after 4 h. The mean particle size of ILH also reduced in PBS, but only to 2.51 ± 0.9 µm, which suggests that the reduction observed was due to aggregates dispersing into individual particles throughout the dispersion period, rather than particle degradation. In contrast, the reduction in ILH particle size observed in ALF, along with the corresponding reduction in DPn, indicates that ILH microparticles are prone to pH-mediated degradation.

**Fig. 4:**
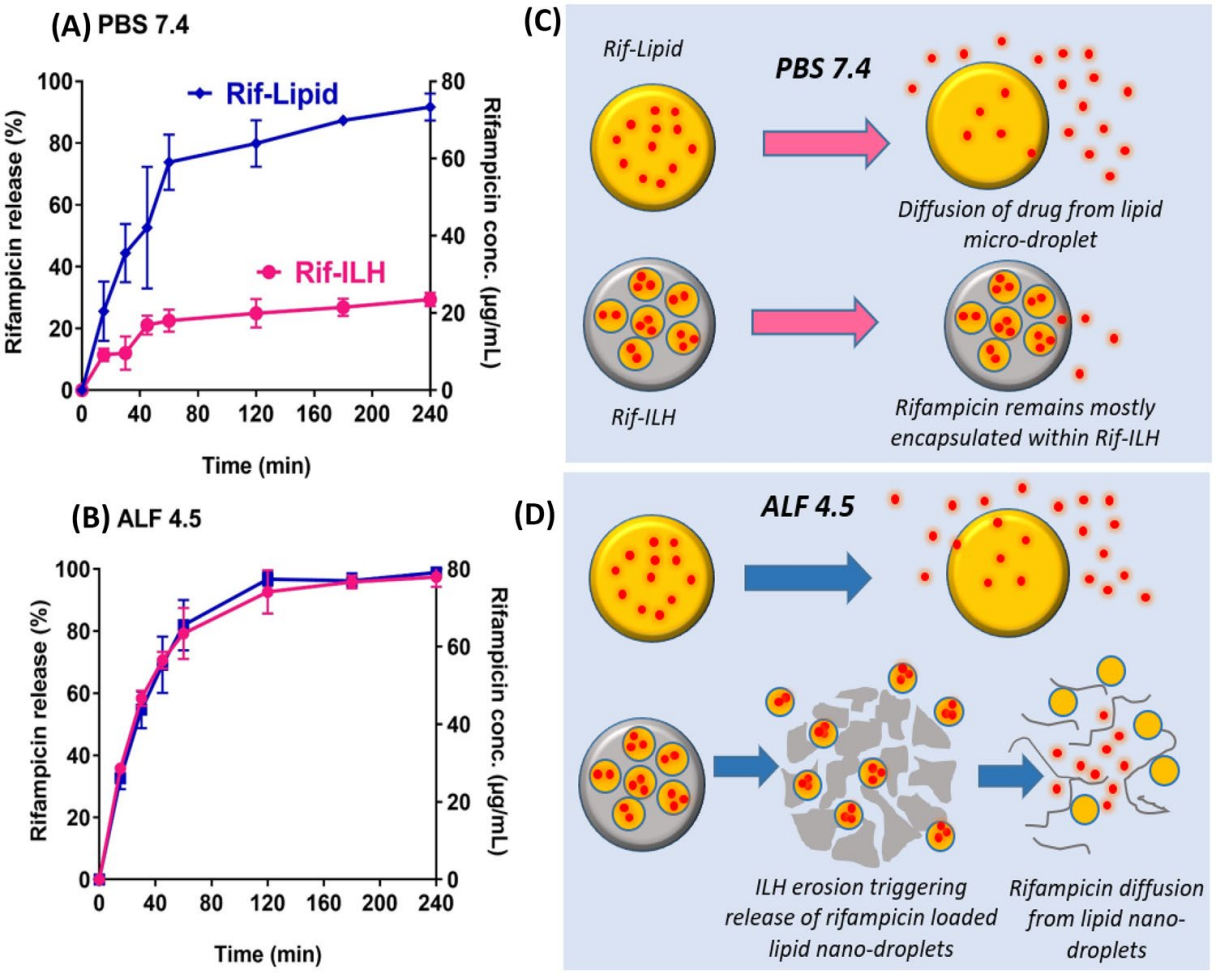
Rifampicin release kinetics from Rif-Lipid (blue) and Rif-ILH microparticles (pink) in **A** neutral media (PBS, pH 7.4, 37 °C) and **B** artificial lysosomal fluid (ALF; pH 4.5, 37 °C). Data is represented as mean ± SD (*n* = 3). **C** Schematic representation illustrating the release behaviour of rifampicin from both Rif-Lipid and Rif-ILH microparticles in **C** neutral media (pH 7.4) and **D** acidic media (pH 4.5)

To validate the pH-responsive release behaviour of rifampicin when encapsulated within ILH microparticles, drug release studies were performed in PBS and ALF, and where release behaviour from Rif-ILH was contrasted against a Rif-Lipid microemulsion of a comparable particle size. In neutral media, only 22.5 ± 3.6% of rifampicin was released from Rif-ILH particles after 1 h, in contrast to 59.0 ± 7.1% for Rif-Lipid (Fig. 4A). Moreover, rifampicin release continued to increase for Rif-Lipid over the 4-h release period, leading to 73.4 ± 3.5% release. Restricted rifampicin release was observed for Rif-ILH microparticles throughout the entire 4-h period, whereby only 29.4 ± 2.2% of rifampicin partitioned towards the aqueous media (Fig. 4), emphasizing the capacity for the inulin three-dimensional matrix of Rif-ILH to protect against drug diffusion into the aqueous media.

The pH-dependent release mechanism of Rif-ILH microparticles was exhibited in ALF at pH 4.5, where over three-quarters of the rifampicin was released within 1 h, with a total release of 79.1 ± 0.2 µg/mL being observed after 4 h. For Rif-Lipid, the change in pH did not induce any significant differences in rifampicin release kinetics (Fig. 4). These findings confirm that the inulin ‘coating’ protects the majority of lipid-encapsulated drug from premature release within plasma-simulating media, due to inulin forming a stable shell surrounding the encapsulated lipid nanoparticles and drug molecules. However, once exposed to acidic ALF media, inulin chains rapidly decompose into oligosaccharide and monosaccharide units, as evidenced through a decrease in DPn and particle size, thus promoting the diffusion of rifampicin from the exposed lipid nano-droplets [11–16]. This is in accordance with previous findings that have shown the pH-provoked release mechanism of drugs conjugated to inulin chains, whereby in neutral media, the drug-inulin conjugate is stable, but once exposed to an environment simulating lysosomal media, the drug is cleaved from the inulin chain due to inulin hydrolysis. One key example is where inulin was used as a carrier for the delivery of the anti-tuberculosis (TB) drug, isoniazid, to monocytes, with the inulin-conjugated isoniazid exhibiting a pH-dependent release with less than 10% release in pH 7.4 and > 40% drug release in pH 4.5 [17].

### Cytotoxicity assessment of ILH microparticles

**Fig. 5:**
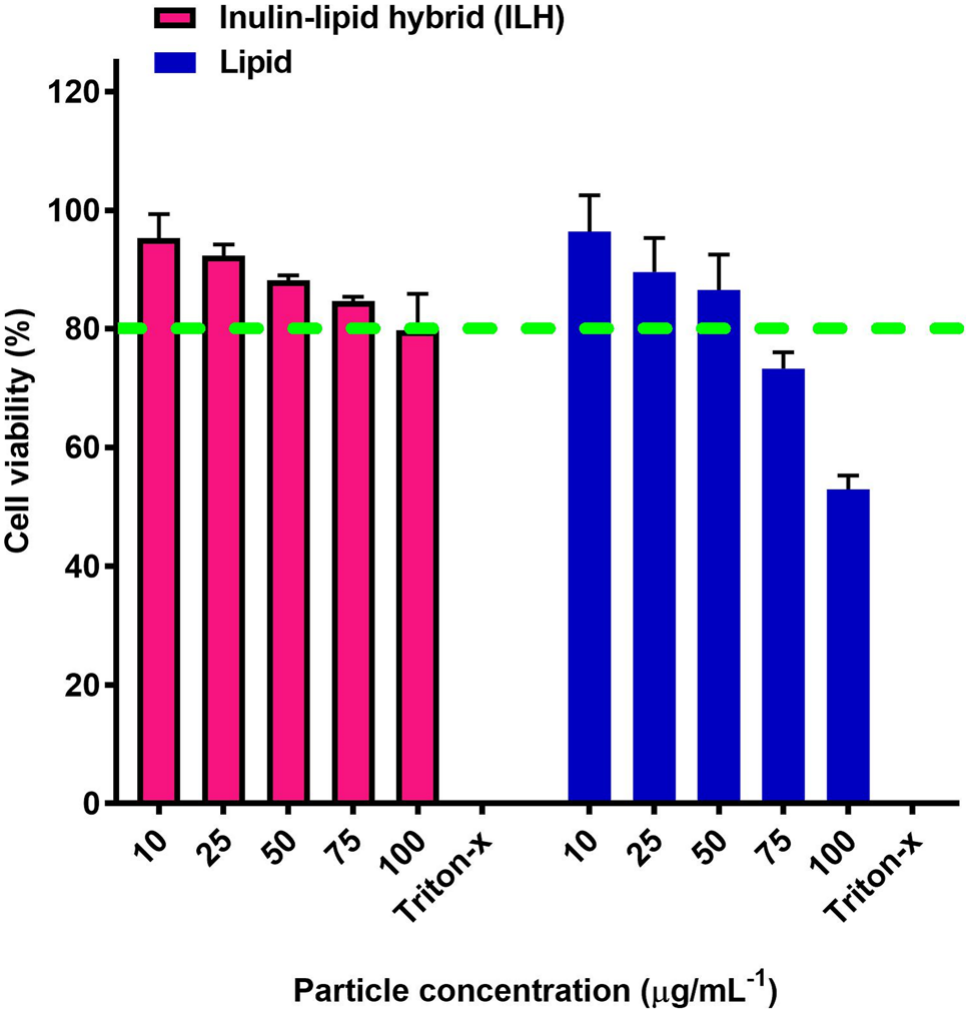
Cellular viability of inulin-lipid hybrid (ILH) microparticles (pink bars) and lipid micro-droplets (blue bars) in RAW 264.7 cells determined via MTT assay (mean ± SD, *n* = 3)

The cytotoxicity of ILH microparticles and lipid micro-droplets was evaluated using the MTT assay in RAW 264.7 macrophages (Fig. 5). Triton-X was used as a positive control with no cellular viability being observed after 1-h incubation. Both ILH microparticles and lipid micro-droplets exhibited a dose-dependent effect on macrophage viability, whereby dose increases from 10 to 100 µg/mL led to a reduction in cellular viability, from 95.2 to 79.7% for ILH and from 96.3 to 52.9% for lipid micro-droplets. Importantly, the presence of the inulin coating for ILH microparticles reduced the cytotoxicity of the particles with the cellular viability remaining ≥ 80% even at 100 µg/mL particle concentration [9]. Subsequently, these findings validate the safety of ILH microparticles towards macrophages. Cellular uptake studies were performed at a particle concentration of 50 µg/mL for both ILH microparticles and lipid micro-droplets, since at this concentration, both systems exerted a cellular viability of > 80%.

### Cellular uptake studies

To understand whether the inulin coating of ILH microparticles promotes cellular uptake compared to uncoated lipid micro-droplets, both formulations were fluorescently labelled with Nile red (lipophilic dye) and the intracellular localization of particles was determined based on differences in fluorescence intensities within the cells, using flow cytometry. Both the cells incubated with Nile red solution as well as control group (no treatment) exhibited no significant difference in fluorescence intensity, thus confirming the inability for the pure dye to promote uptake. Subsequently, any enhancement in Nile red intensity within the cells treated with Nile red particles can be attributed to particle uptake.

**Fig. 6:**
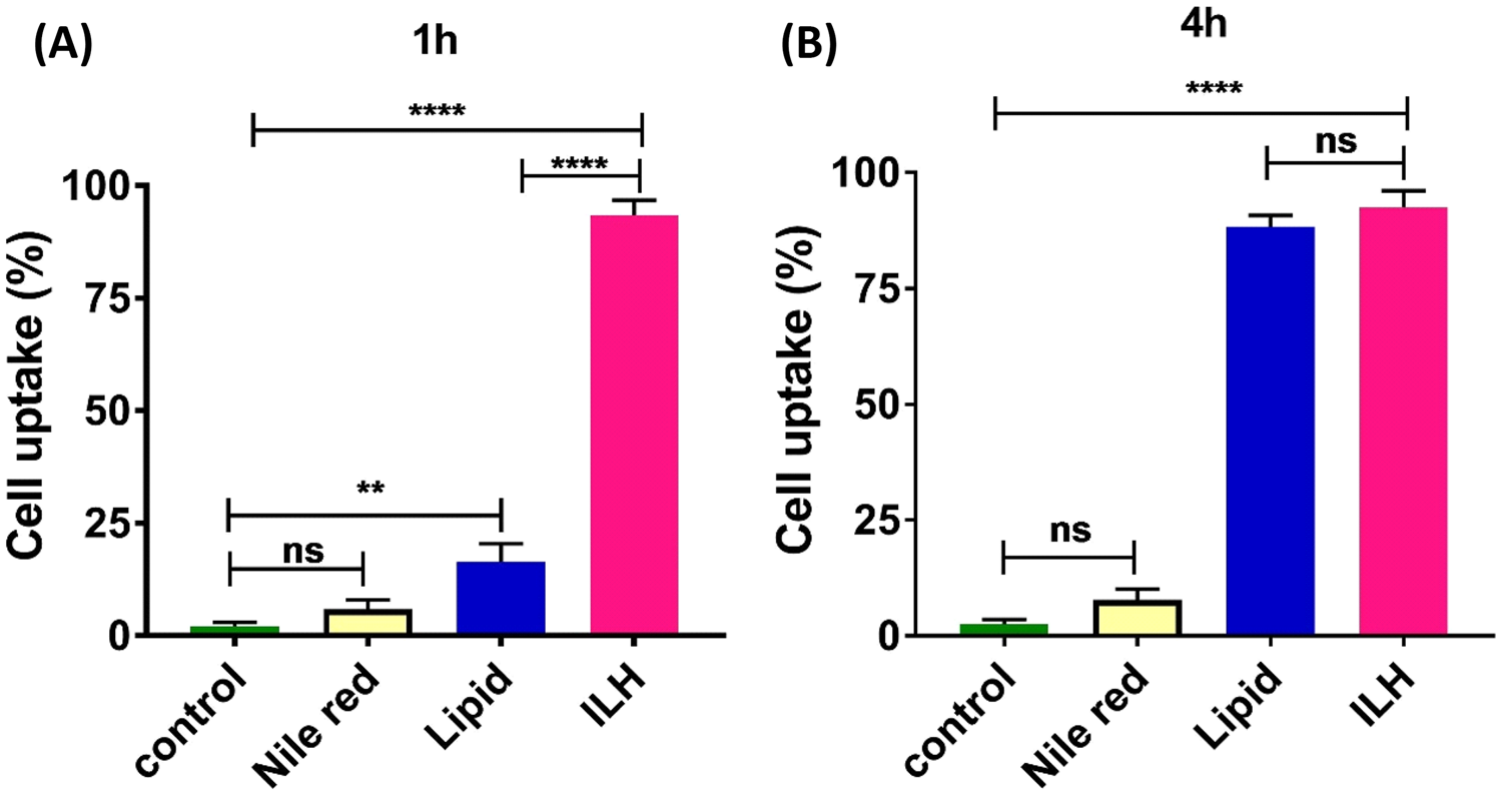
Cellular uptake of lipid micro-droplets (blue bars) and ILH microparticles (pink bars) loaded with Nile red in RAW 264.7 cells after **A** 1-h and **B** 4-h incubation. No significant difference was observed between cells treated with Nile red (yellow bars) compared to the control group (no treatment; green bars)

**Fig. 7:**
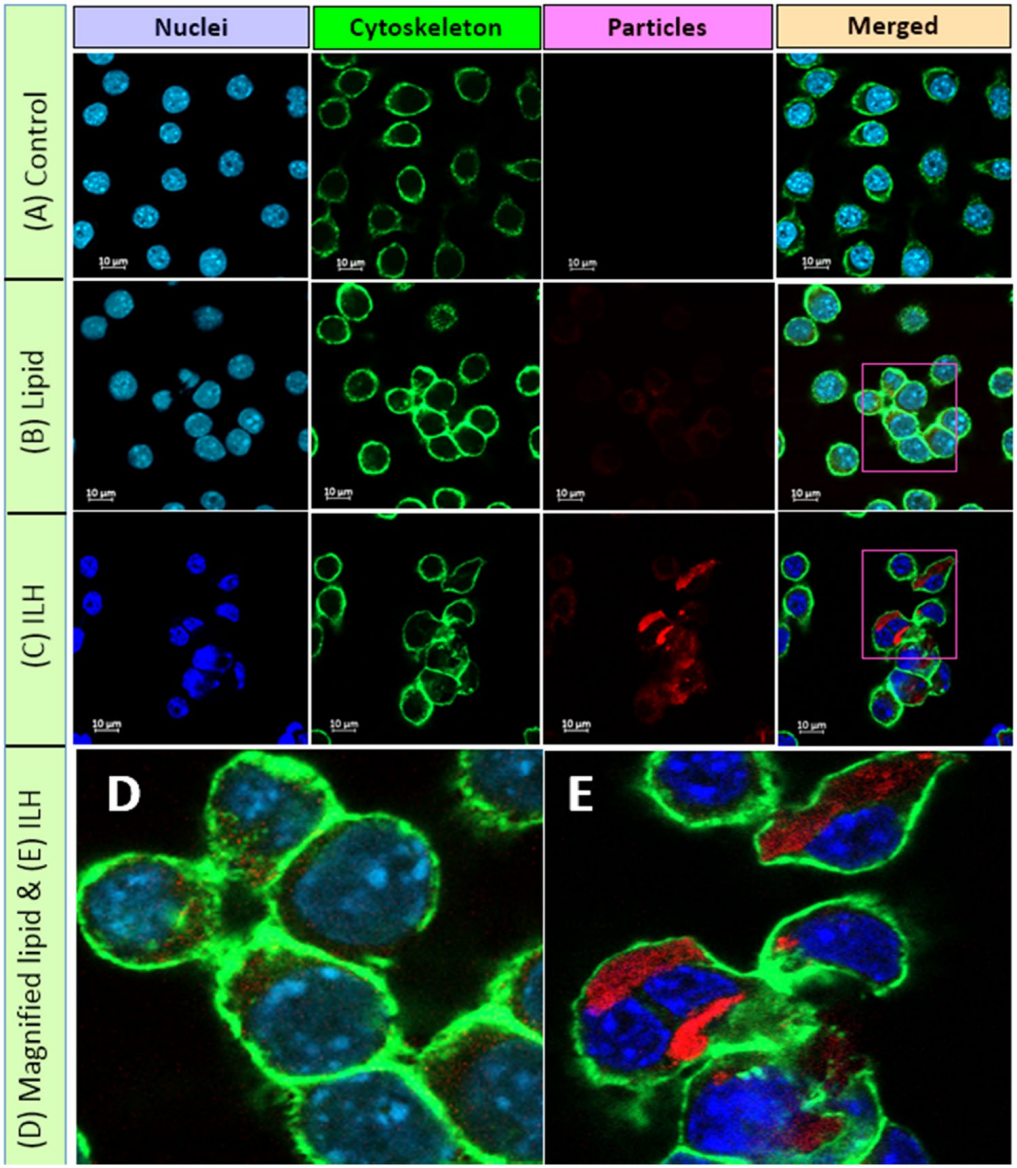
Confocal microscopy images for the uptake of Nile red formulations by RAW 264.7 macrophages, after 1-h incubation with **A** Nile red solution, **B** Nile red–loaded lipid microparticles and **C** Nile red–loaded ILH microparticles. Magnified regions of **D** Nile red–loaded lipid micro-droplets and **E** Nile red–loaded ILH microparticles. All formulations were dosed at an equivalent dye concentration of 50 µg/mL. Nuclei were stained with DAPI dye (blue region); the cellular cytoskeleton was stained with Alexa-488 (green region); and the particles were stained with Nile red (red region)

Incubation with both lipid micro-droplets and ILH microparticles triggered cellular uptake of Nile red, with 16.4 ± 4.0% and 93.4 ± 3.4% particles being internalized after 1 h, respectively (Fig. 6). Thus, the degree of particle uptake for ILH microparticles was 6- to 16-fold greater compared to the lipid micro-droplet and the pure Nile red solution after 1-h incubation. This was further evidenced using confocal microscopy, whereby there is enhanced dye internalization for ILH particles after 1-h incubation, compared to lipid micro-droplets and pure dye (Fig. 7). No additional uptake was observed for ILH microparticles between 1 and 4 h. In contrast, lipid micro-droplets continued being internalized within macrophages during this period, achieving a total of 88.3 ± 2.5% uptake after 4 h.

Similar outcomes were reported in earlier studies where inulin-based drug delivery systems were used for targeted intracellular antibiotic delivery to cells infected with intracellular pathogens, e.g. *Mycobacterium tuberculosis* [29, 38]. The results suggested the ability of inulin to promote a rapid uptake of particles by infected cells through endocytosis pathways; however. the specific receptor(s) mediating this process are unknown [23, 39, 40]. Ultimately, it is suggested that incorporation of inulin facilitated the rapid endocytosis of ILH microparticles by phagocytic cells, i.e. RAW 264.7 cells, leading to higher uptake of ILH microparticles compared to lipid micro-droplets in a shorter period of time. For both lipid micro-droplets and ILH microparticles, the particle size could be considered ideal for cellular uptake, since previous studies have highlighted the enhanced capacity for particles with intermediate particles size (~ 2–3 μm in diameter) to be engulfed by macrophages, compared to submicron particles and particles exceeding 10 µm [41]. Subsequently, it was considered that the particle size of ILH microparticles synthesized in this study was ideal for promoting cellular uptake; however, further optimization may lead to production of a more uniform particle size distribution and therefore greater enhancements in particle internalization within macrophages in key target tissues associated with intracellular infection, such as the lungs.

### Rifampicin delivery to macrophages

**Fig. 8:**
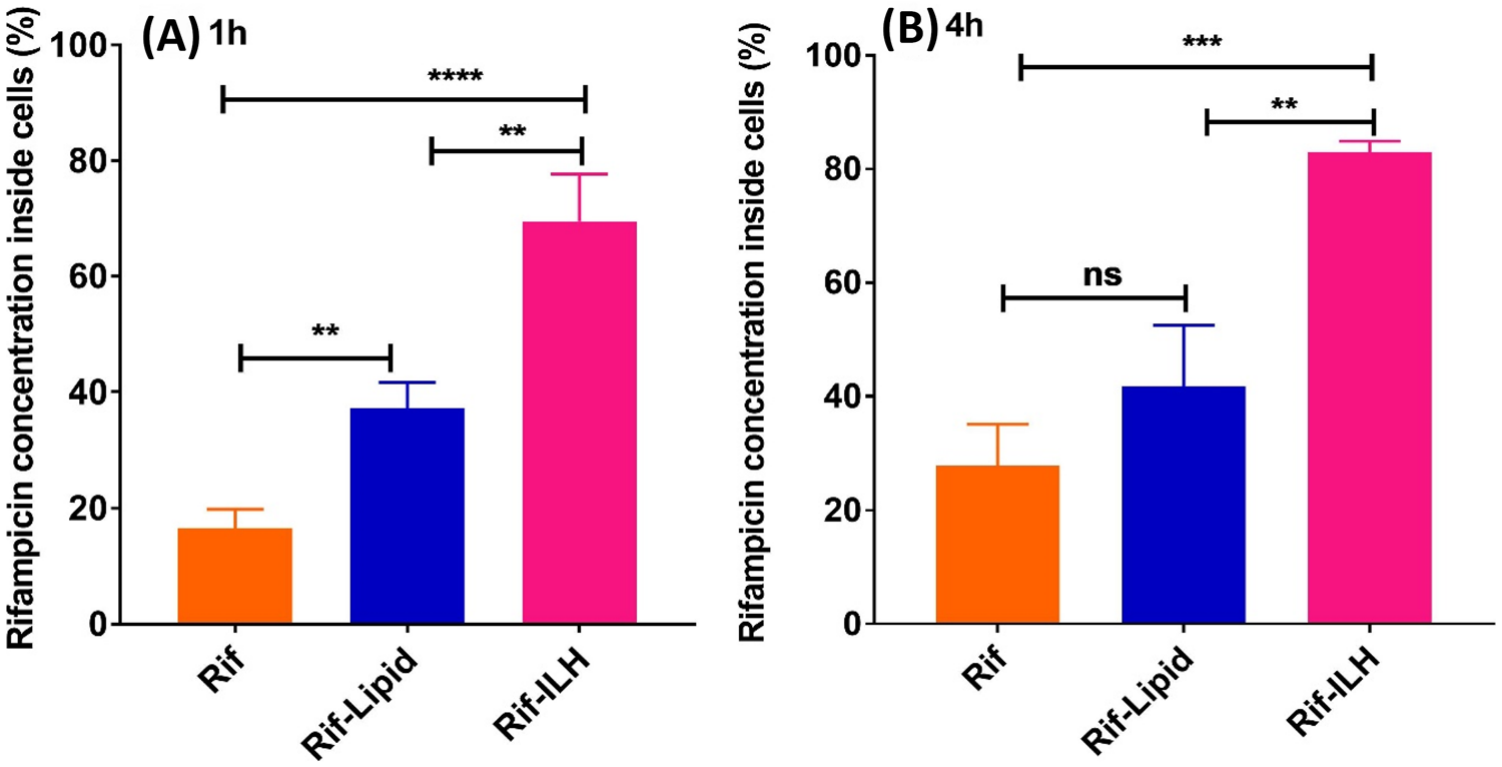
Internal rifampicin concentration within macrophages when incubated with a rifampicin solution (orange bars), Rif-Lipid (blue bars) and Rif-ILH microparticles (pink bars), when dosed at a rifampicin concentration 50 µg/mL after **A** 1-h and **B** 4-h incubation time (mean ± SD, *n* = 3)

The degree of rifampicin internalization within macrophages was examined by incubating RAW 264.7 cells with a rifampicin solution, rifampicin-loaded ILH (Rif-ILH) microparticles and lipid micro-droplets (Rif-Lipid) (Fig. 8). After 1-h incubation, 37.3 ± 4.3% and 69.5 ± 8.1% of the rifampicin dose was detected inside the macrophages when treated with Rif-Lipid and Rif-ILH microparticles, respectively, compared to 16.5 ± 3.3% for the cells treated with pure rifampicin solution. As the incubation time increased from 1 to 4 h, the degree of rifampicin uptake when encapsulated within Rif-Lipid and Rif-ILH microparticles did not increase, indicating that rifampicin was internalized within the first hour of incubation. Furthermore, there was no significant difference in intracellular rifampicin concentration for the cells treated with Rif-Lipid compared to the pure drug solution after 4-h incubation, which did not correlate with the cellular uptake data obtained for the Nile red lipid micro-droplets. This can be attributed to the inability for lipid micro-droplets to protect the encapsulated cargo (rapid partitioning into solution), specifically rifampicin, in neutral conditions equivalent to the extracellular environment. That is, drug release data indicated that over 75% of rifampicin is released from the lipid micro-droplet after 1 h at pH 7.4 (Fig. 4). Thus, after this period, lipid micro-droplets cannot facilitate uptake of rifampicin. In contrast, Rif-ILH microparticles protect the encapsulated rifampicin from premature release in the extracellular environment, until phagocytosed by the macrophages, where the acidic lysosomal media hydrolyses inulin (Fig. 3), triggering the release of rifampicin. This highlights the important role of the pH-triggered release mechanism of Rif-ILH microparticles in transporting rifampicin to macrophages.

### Antibacterial efficacy of rifampicin formulations against SCV *S. aureus*

**Fig. 9:**
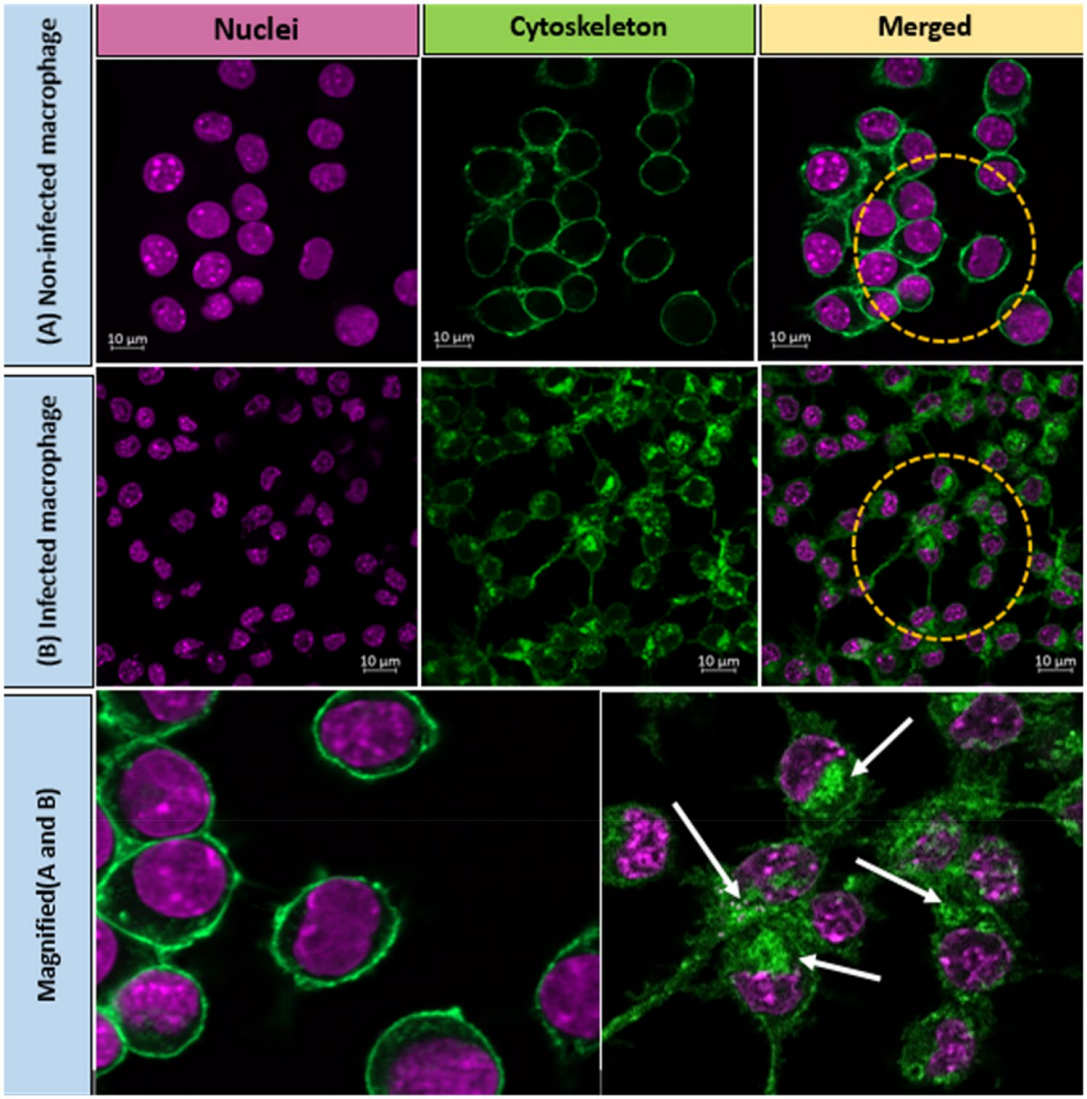
The intracellular presence of SCV *S. aureus* as observed using confocal microscopy. The nuclei and the cell membrane of macrophages were shown as purple and green, respectively. The localized aggregation of dye associated with the bacterial cell wall within the cells (as indicated by white arrows) demonstrates the presence of SCV *S. aureus* within cells

RAW 264.7 cells were infected with SCV *S. aureus* as a model intracellular pathogen. This pathogen is responsible for unresolved clinical problems associated with chronic infections and owing to their slow metabolism, SCV *S. aureus* are phenotypically smaller compared to the parent strain of *S. aureus* [35]*.* Here, the intracellular localization of SCV *S. aureus* within RAW 264.7 cells was visually confirmed using confocal microscope. As illustrated in Fig. 9, the cytoskeleton of both non-infected and infected macrophages was stained green with deep purple nuclei, while the aggregation of dye (green spots) was associated with bacterial cell wall stained with Alexa 488 and highlighted the presence of SCV *S. aureus* within the cells.

**Fig. 10:**
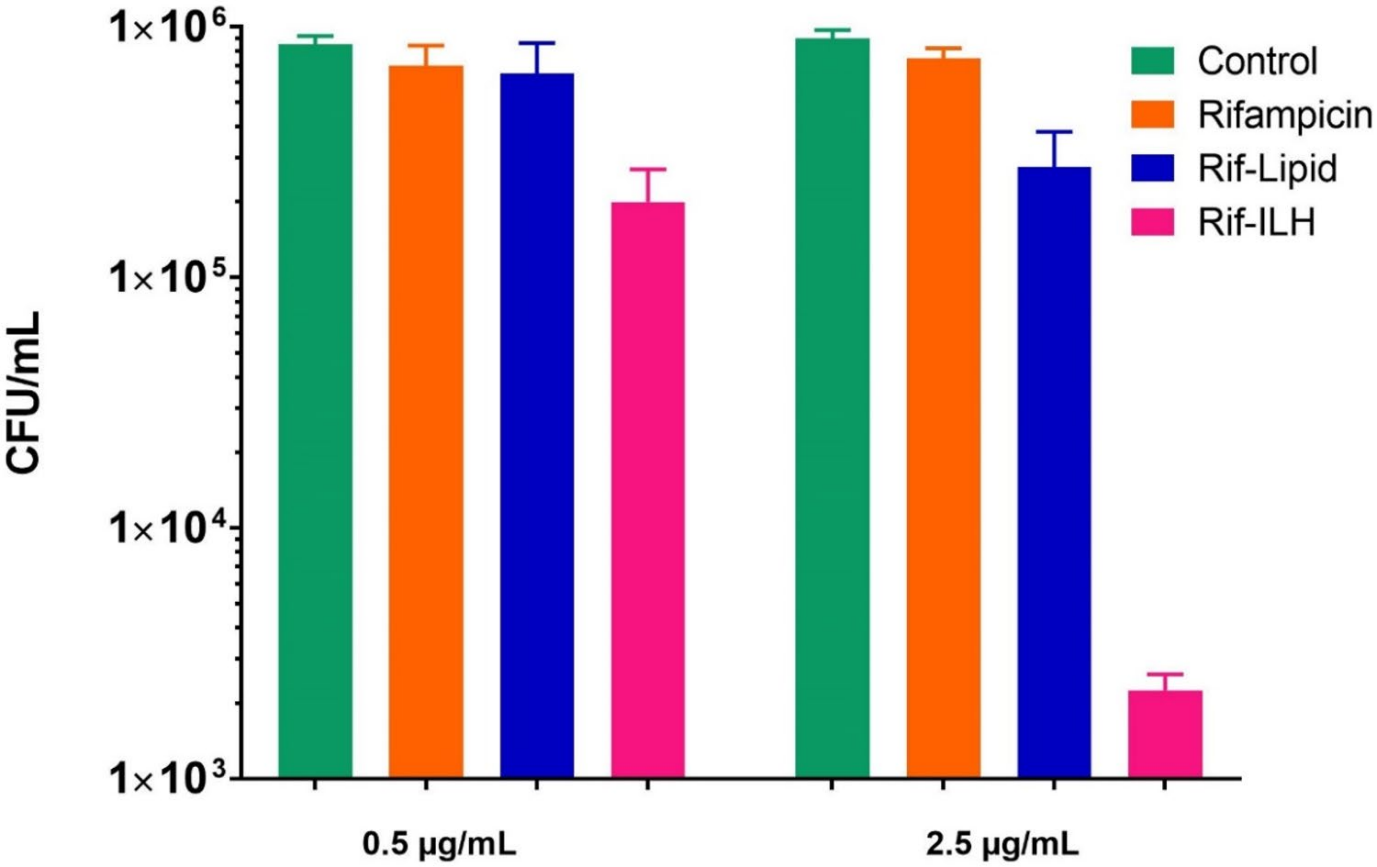
Efficacy of rifampicin-loaded formulations on the quantitative reduction of intracellular SCV *S. aureus*

The selection of rifampicin concentrations to evaluate the efficacy of formulations was based on the Minimum Inhibitory Concentration (MIC) against SCV *S. aureus* [34], which was previously determined to be 0.125 µg/mL [16]. Subsequently, a rifampicin concentration equivalent to 4 × MIC (0.5 µg/mL) was initially selected to ascertain the efficacy of each rifampicin formulation in triggering a reduction in intracellular bacteria. Following the 4-h treatment with rifampicin solution and Rif-Lipid, SCV *S. aureus*–infected macrophages exhibited a negligible reduction in CFU of SCV *S. aureus*, which was shown to be statistically comparable to the control group (i.e. no treatment) (Fig. 10). The poor ability of unformulated rifampicin to significantly reduce the abundance of viable bacteria was anticipated since uptake studies revealed the inability for rifampicin to be internalized within macrophages. For Rif-Lipid formulation, premature drug release in neutral media reduced the exposure of intracellular pathogens to the antibiotic drug, and thus, Rif-Lipid were unable to significantly improve the overall efficacy of rifampicin at 0.5 µg/mL. In contrast, treatment of infected macrophages with Rif-ILH resulted in a significant improvement in the antibacterial activity of rifampicin against SCV *S. aureus*. Rif-ILH demonstrated a ~ 4-log greater reduction in CFU compared to the rifampicin solution and Rif-Lipid. This correlates well with the macrophage uptake findings, where the intracellular concentration of rifampicin when hosted within Rif-ILH was over 2-log greater, compared to that within pure rifampicin and Rif-lipid. This further confirmed the increased intracellular concentration of the drug within the vicinity of the target site (i.e. site of pathogen confinement) and therefore facilitated more efficient bacterial killing.

To evaluate a dose-dependent effect of rifampicin-loaded formulations on antibacterial activity, infected macrophages were treated with a rifampicin dose 20-fold greater than its MIC (i.e. 2.5 μg/mL) (Fig. 10). Increasing the rifampicin dose from 4 × MIC to 20 × MIC did not provide any further improvement in bacterial reduction for Rif-Lipid (i.e. the CFU reduced from 6.5 × 10^5^ CFU/mL to 2.7 × 10^5^ CFU/mL), while Rif-ILH microparticles showed the greatest antibacterial activity, with a ~ 2-log reduction in CFU (i.e. 2 × 10^5^ CFU/mL to ~ 2.2 × 10^3^ CFU/mL). This is in agreement with the previous study where the maximum efficacy was achieved when SCV *S. aureus*–infected macrophages were treatment with rifampicin-loaded formulations equivalent to 2.50 μg/mL rifampicin dose [34].

Subsequently, this study reveals a new approach for enhancing the antibacterial efficacy of rifampicin against pathogens that shield themselves in intracellular environment of the host, through encapsulation within an inulin-based drug delivery system. Moreover, for the first time, utilizing the ILH carrier as a micro-encapsulation device enabled the advantageous effects of inulin as a bioactive material and delivery vehicle to be harnessed, while eliminating the need for chemical modifications to the drug via a covalent attachment mechanism. To facilitate clinical translation of this new drug delivery approach, further preclinical investigation and validation are necessary. Specifically, the efficacy of this treatment must be demonstrated across broader *S. aureus* strains with greater genetic diversity and in host cells that are not naturally phagocytic but are still capable of harbouring pathogens (e.g. epithelial cells). Furthermore, treatment efficacy must be clearly demonstrated within in vivo studies using validated intracellular models of infection. It is anticipated that the clinical application of micro-encapsulated antibiotics, using ILH, will reduce the high clinical dose required for effective bacterial killing and subsequently reduce severe side effects and toxicity associated with antibiotics’ over-consumption. Owing to the small particle size and low toxicity of ILH, it is anticipated that clinical administration through systemic (e.g. intravenous injection) or pulmonary (e.g. inhalation) routes will provide a novel approach for more effectively and safely treating systemic and lung infections. Previous studies have demonstrated the clinical translatability of biocompatible microparticles in treating such infections, including tuberculosis, and therefore, ILH is well-positioned for further preclinical investigation and optimization to promote translation to the clinic.

## Conclusions

A novel and efficient drug delivery system, based on hybrid microparticles of lipid and inulin, has been developed and validated for its high potential for treatment of macrophages infected with intracellular SCV *S. aureus* pathogens. The synthesized hybrid microparticles demonstrated a pH-responsive release of rifampicin at acidic pH in artificial lysosomal fluid media, with significantly less release in neutral media, which was shown to be crucial in effectively treating pathogens located within the intracellular environment of host cells. Moreover, the inulin-lipid hybrid (ILH) microparticles were internalized efficiently by macrophages and subsequently result in the enhanced intracellular release of rifampicin where bacteria are localized. The ability of the ILH system to deliver the encapsulated cargo intracellularly while avoiding the undesirable drug release prior to uptake by macrophages was shown to be fundamental in promoting a 2-log (100-fold) improvement in antibacterial efficacy.

## Data Availability

The datasets generated during and/or analyzed during the current study are available from the corresponding author on reasonable request.
